# Supplementary material for: Identification of iridoid synthases from Nepeta species: Iridoid cyclization does not determine nepetalactone stereochemistry
Source: Phytochemistry. 2018 Jan;145:48–56. doi: 10.1016/j.phytochem.2017.10.004 (PMC5739345; doi:10.1016/j.phytochem.2017.10.004)

**Supplementary materials and methods for:**

# Identification of iridoid synthases from *Nepeta* species: Iridoid cyclization does not determine nepetalactone stereochemistry

Nathaniel H. Sherden[1], Benjamin Lichman[1], Lorenzo Caputi[1], Dongyan Zhao[2], Mohamed O. Kamileen[1], C. Robin Buell[2], Sarah E. O'Connor[1]*

## Supplementary Figure. $^1$H NMR spectra

8-oxogeranial

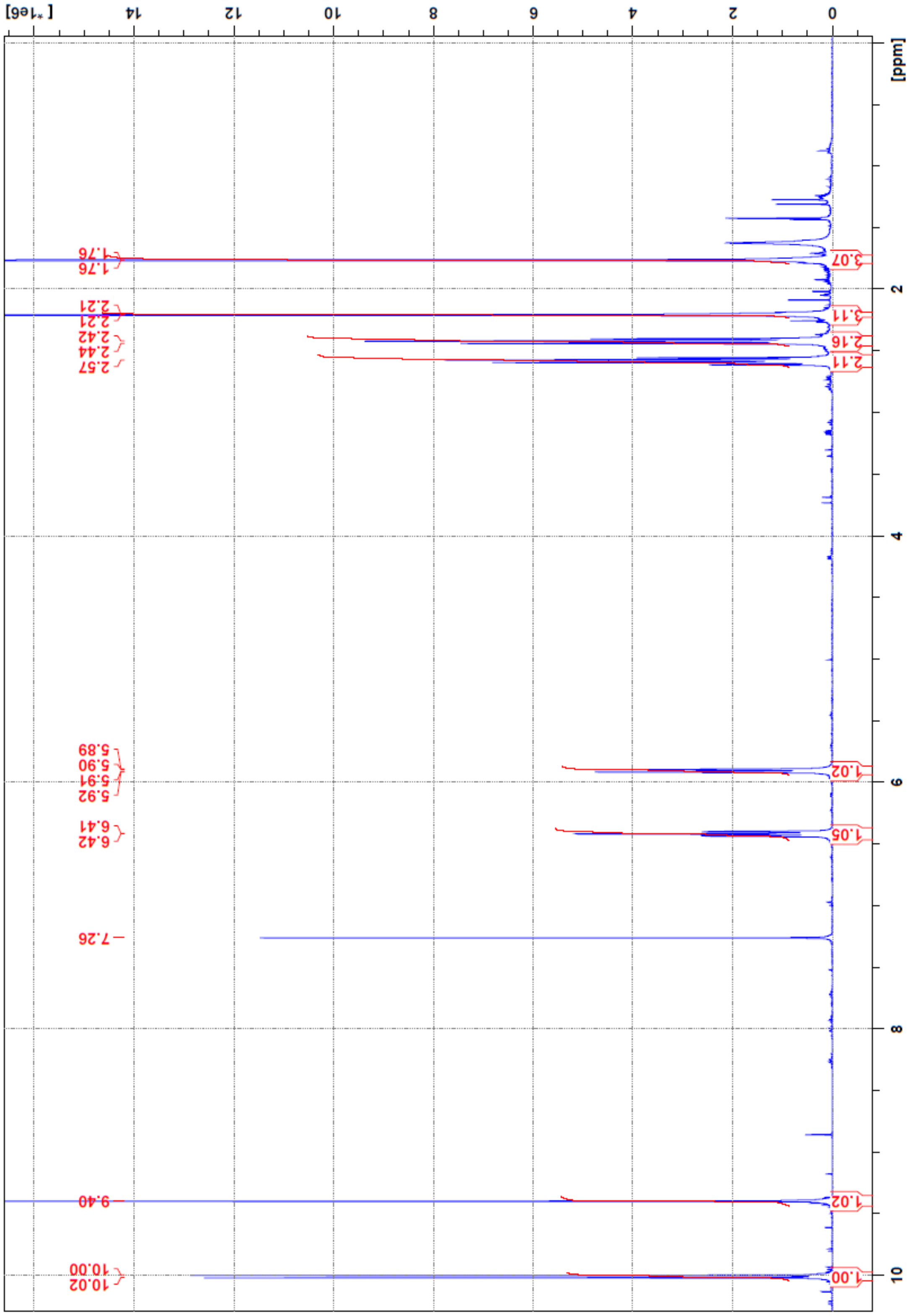

*Cis*-*trans*-nepetalactol

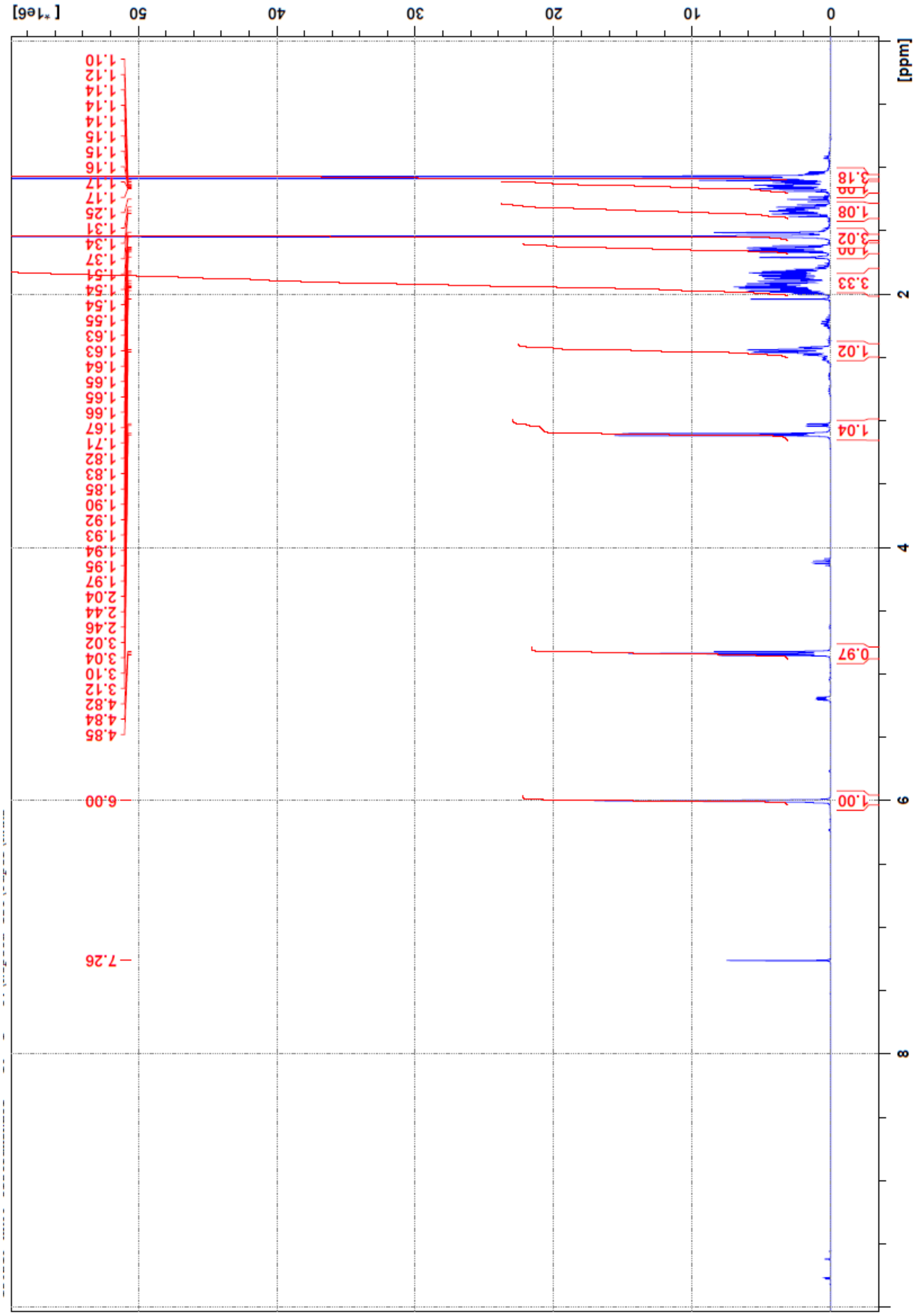

*Cis*-*trans*-nepetalactone

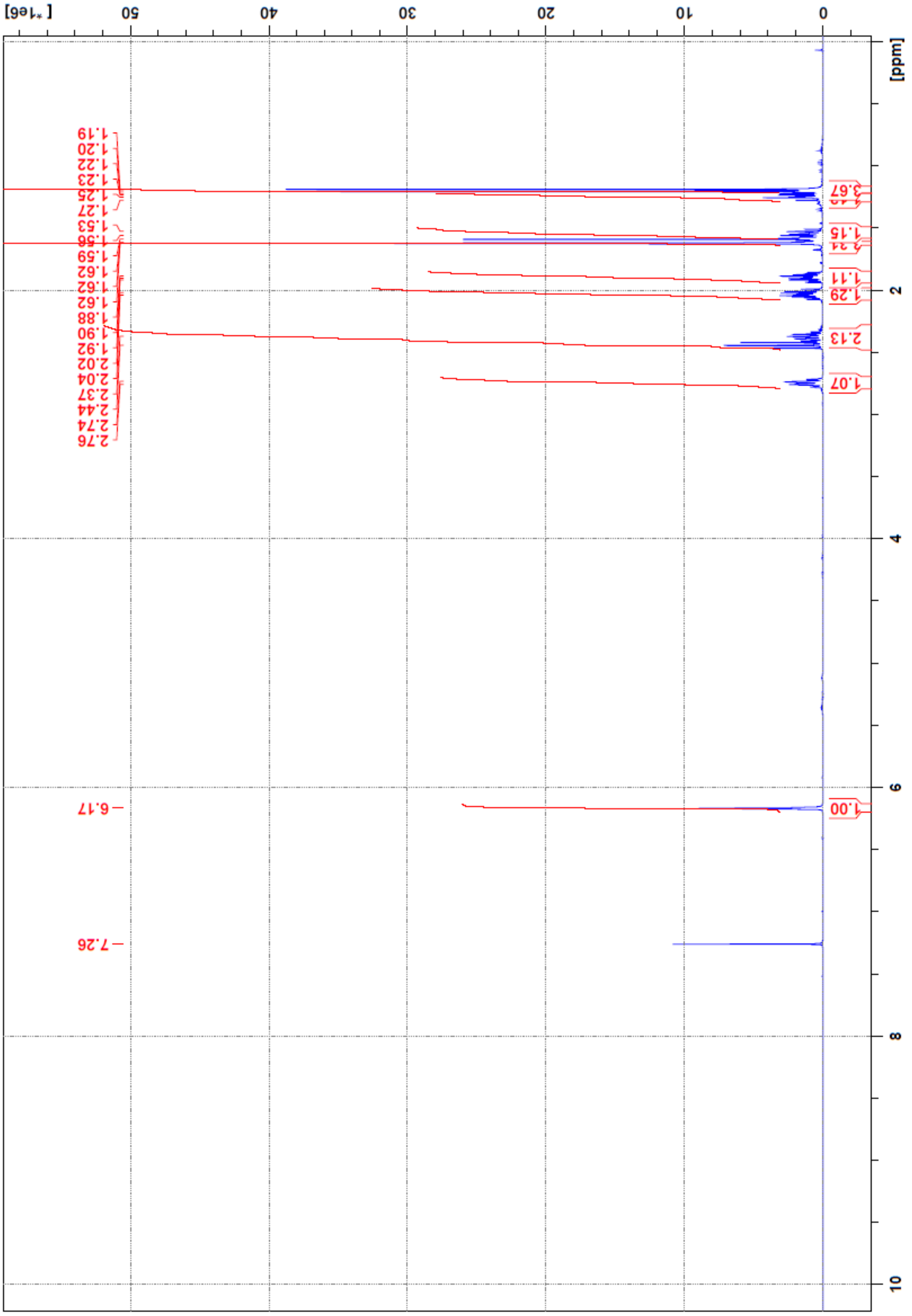

*Trans*-*cis*-nepetalactone

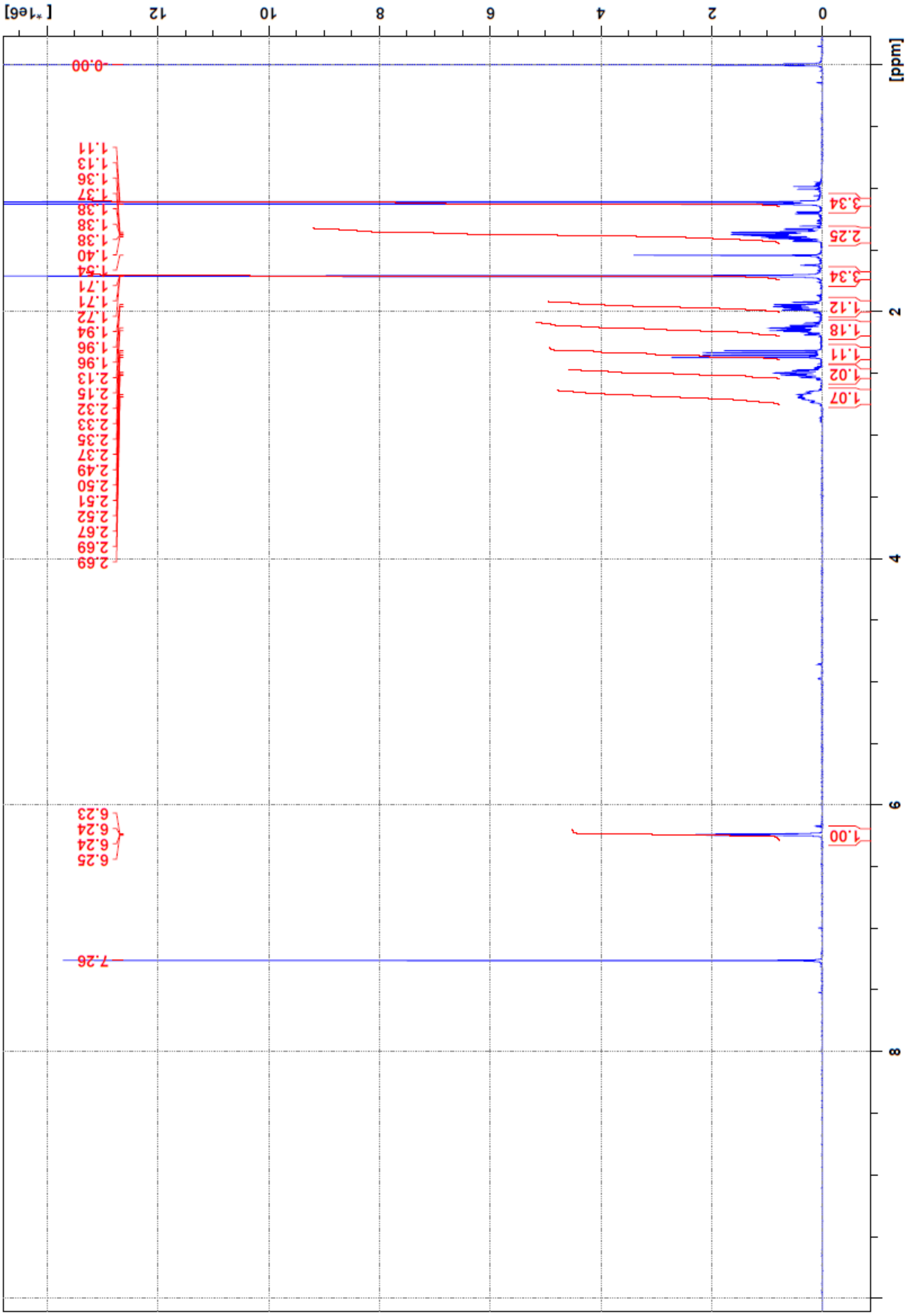

*Trans*-*cis*-iridodial 1H

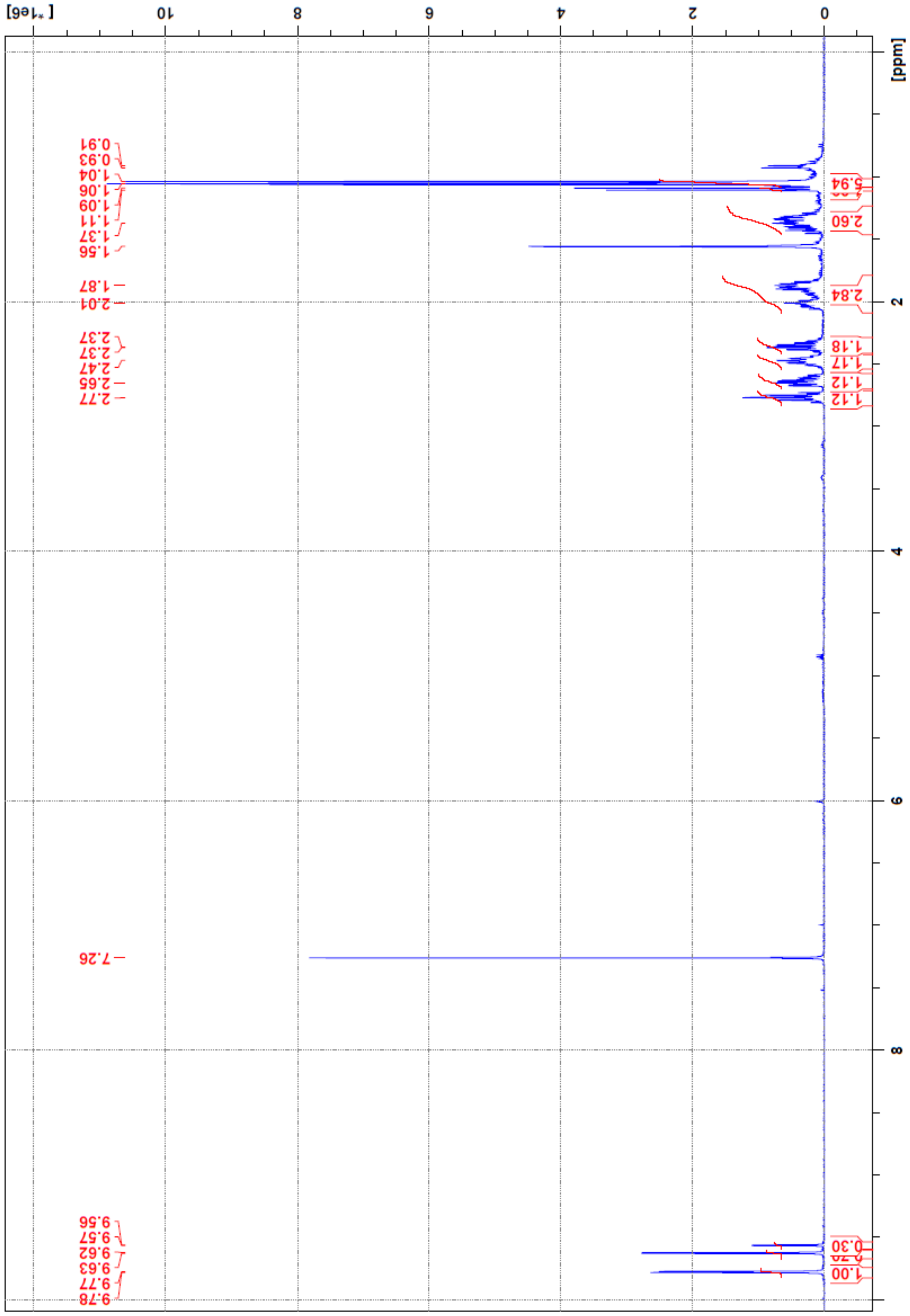

Supplement: mmc1 [file mmc1.pdf]
